# Supplementary material for: Range-wide differential adaptation and genomic offset in critically endangered Asian rosewoods
Source: Proc Natl Acad Sci U S A. 2023 Aug 7;120(33):e2301603120. doi: 10.1073/pnas.2301603120 (PMC10438386; doi:10.1073/pnas.2301603120)

Supplementary Information

Range-wide differential adaptation and genomic offset in critically endangered Asian rosewoods

Tin Hang Hung[1,*], Thea So[2], Bansa Thammavong[3], Voradol Chamchumroon[4], Ida Theilade[5], Chhang Phourin[2], Somsanith Bouamanivong[6], Ida Hartvig[7,8], Hannes Gaisberger[9,10], Riina Jalonen[11], David H. Boshier[1], John J. MacKay[1,*]

1. Department of Biology, University of Oxford, Oxford OX1 3RB, United Kingdom
2. Institute of Forest and Wildlife Research and Development, Phnom Penh, Cambodia
3. National Agriculture and Forestry Research Institute, Forestry Research Center, Vientiane, Laos
4. The Forest Herbarium, Department of National Park, Wildlife and Plant Conservation, Ministry of Natural Resources and Environment, Bangkok, Thailand
5. Department of Food and Resource Economics, Faculty of Science, University of Copenhagen, Denmark
6. National Herbarium of Laos, Biotechnology and Ecology Institute, Ministry of Science and Technology, Vientiane, Laos
7. Forest Genetics and Diversity, Department of Geosciences and Natural Resource Management, University of Copenhagen, Denmark
8. Center for Evolutionary Hologenomics, Globe Institute, University of Copenhagen, Denmark
9. Bioversity International, Rome, Italy
10. Paris Lodron University, Salzburg, Austria
11. Bioversity International, Serdang, Malaysia

Corresponding authors:

*T.H.H.: tin-hang.hung@biology.ox.ac.uk; *J.J.M.: john.mackay@biology.ox.ac.uk

Supplementary Table 1.

Assembly statistics of the *D. cochinchinensis* genome (Dacoc_1.4).

Assembly identifier	Dacoc_1.4
Species	*Dalbergia cochinchinensis* Pierre ex Laness
NCBI taxonomy ID	106130

	Hi-C reads	***Contigs***	***Primary haplotigs***	***Scaffolds***
Total size (Mbp)	54,973.60	1,353.09	621.01	621.01
Number of sequences	366,490,675	6,443	1,238	512
Average length of sequences	150	210,008.8	1,003,247.1	1,212,910.1
N50	150	1,345,847	21,352,153	59,971,594

Supplementary Table 2.

Statistics of Hi-C library prepared with the restriction enzyme DpnII and proximity-ligated.

Total read pairs (RPs)	366,490,675
RP length	150 bp
High quality (HQ) RPs	25.37%
RPs > 10 Kb apart	6.82%
RPs > 10 Kb apart (CTGs > 10 Kb)	10.37%
Intercontig RPs	33.22%
Intercontig hig-quality (HQ) RPs	6.31%
Same strand RPs	7.22%
Split reads	13.53%
Duplicate reads (extrapolated)	8.72%
Genome scaffolding sufficiency	
Same-strand high-quality* (HQ) read pairs (RPs)	5.03%
Informative RPs**	5.21%

Supplementary Table 3.

Assembly statistics of the *D. oliveri* genome (Daoli_0.3).

Assembly identifier	Daoli_0.3
Species	*Dalbergia oliveri* Gamble ex Prain
NCBI taxonomy ID	1030150

	Pore-C reads	***Contigs***	***Primary haplotigs***	***Scaffolds***
Total size (Mbp)	13,462.40	814.69	687.92	689.25
Number of sequences	3,098,972	3,249	2,239	2,977
Average length of sequences	4,344.2	250,750	307,244.3	231,525
N50	5,789	474,015	623,924	38,426,337

Supplementary Figure 1.

Snailplot of the genome assembly (a) Dacoc_1.4 and (b) Daoli_0.3 showing scaffold statistics, BUSCO statistics, and assembly base composition.

(a)

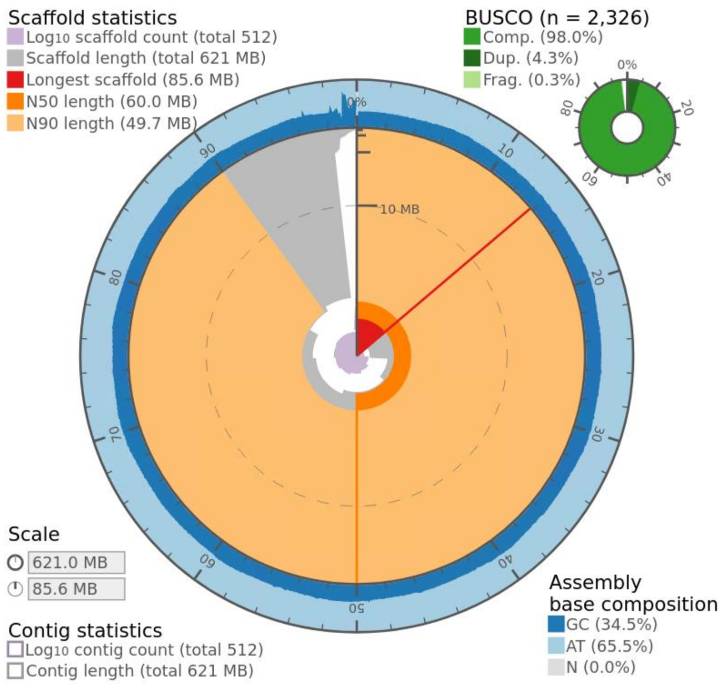

(b)

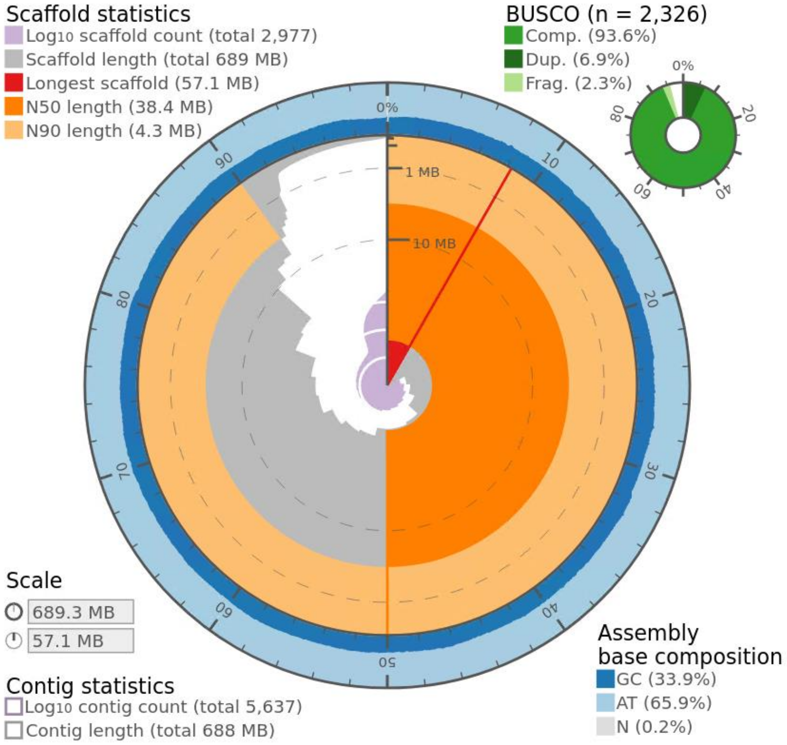

Supplementary Table 4.

Composition and percentage of the repeat families in the final genome of Dacoc_1.4 identified with RepeatModeler and LTRharvest.

	**Number of elements**	**Total length (bp)**	**Percentage of sequence**
**Dacoc_1.2**		621009970	
**Bases masked**		402405433	64.80
**Retroelements**	405732	293464285	47.26
SINEs	0	0	0.00
Penelope	0	0	0.00
LINEs	9518	3856482	0.62
CRE/SLACS	0	0	0.00
L2/CR1/Rex	0	0	0.00
R1/LOA/Jockey	0	0	0.00
R2/R4/NeSL	0	0	0.00
RTE/Bov-B	3633	803221	0.13
L1/CIN4	5885	3053261	0.49
LTR elements	396214	289607803	46.63
BEL/Pao	0	0	0.00
Ty1/Copia	209546	94712214	15.25
Gypsy/DIRS1	176990	189459322	30.51
Retroviral	0	0	0.00
**DNA transposons**	11462	6635025	1.07
hobo-Activator	1830	871791	0.14
Tc1-IS630-Pogo	0	0	0.00
En-Spm	0	0	0.00
MuDR-IS905	0	0	0.00
PiggyBac	0	0	0.00
Tourist/Harbinger	3272	1401349	0.23
Other (Mirage, P-element, Transib)	0	0	0.00
**Rolling-circles**	3613	2108774	0.34
**Unclassified**	359305	90585676	14.59
Total interspersed repeats		390684986	62.91
Simple repeats	131568	8474809	1.36
Low complexity	23616	1136864	0.18

Supplementary Table 5.

Composition and percentage of the repeat families in the final genome of Daoli_0.3 identified with RepeatModeler and LTRharvest.

	**Number of elements**	**Total length (bp)**	**Percentage of sequence**
**Retroelements**	455479	339092238	49.2
SINEs	0	0	0
Penelope	0	0	0
LINEs	10758	4445290	0.64
CRE/SLACS	0	0	0
L2/CR1/Rex	0	0	0
R1/LOA/Jockey	0	0	0
R2/R4/NeSL	0	0	0
RTE/Bov-B	3783	973287	0.14
L1/CIN4	6975	3472003	0.5
LTR elements	444721	334646948	48.55
BEL/Pao	0	0	0
Ty1/Copia	229457	108551031	15.75
Gypsy/DIRS1	200968	220263371	31.96
Retroviral	181	91342	0.01
**DNA transposons**	16939	12338687	1.89
hobo-Activator	2978	1273955	0.18
Tc1-IS630-Pogo	1440	179489	0.03
En-Spm	0	0	0
MuDR-IS905	0	0	0
PiggyBac	0	0	0
Tourist/Harbinger	2277	1185567	0.17
Other (Mirage, P-element, Transib)	0	0	0
**Rolling-circles**	5146	3333791	0.48
**Unclassified**	376428	91174602	13.23
Total interspersed repeats	1757530	442605527	64.22
Simple repeats	143462	5735706	0.83
Low complexity	25174	1218960	0.18

Supplementary Table 6.

Mapping statistics of RNA-seq raw reads from a previous experiment using pooled sample_from leaves, stems, and roots of *D. cochinchinensis* and *D. oliveri* on the Dacoc_1.4 and Daoli_0.3 genomes respectively.

	Dacoc_1.4	Daoli_0.3
Number of input reads	135,924,672	132,696,766
Average input read length	299	299
	Unique reads	
Number of uniquely mapped reads	115,815,104 (85.21%)	122,215,722 (92.10%)
Average mapped length	294.91	296.69
Number of splices<br>GT/AG<br>GC/AG<br>AT/AC<br>Non-canonical	86,392,015<br>84,315,981<br>956,359<br>47,749<br>1,071,926	96,847,851<br>95,280,260<br>1,148,136<br>44,025<br>375,430
Mismatch rate per base (%)	0.86	2.87
Deletion rate per base (%)	0.05	0.03
Deletion average length	2.95	2.87
Insertion rate per base (%)	0.11	0.03
Insertion average length	1.62	2.50
	Multi-mapping reads	
Number of reads mapped to multiple loci	11,182,606 (8.23%)	4,875,679 (3.67%)
Number of reads mapped to too many loci	27,541 (0.02%)	230,932 (0.17%)
	Unmapped reads	
Number of reads with too many mismatches	0 (0.00%)	0 (0.00%)
Number of reads too short	8,890,737 (6.54%)	4,954,750 (3.73%)
Number of other reads	8,684 (0.01%)	419,683 (0.32%)
	Chimeric reads	
Number of chimeric reads	0 (0.00%)	0 (0.00%)

Supplementary Figure 2.

Cumulative frequency of gene models of **(a)** Dacoc_1.4 and **(b)** Daoli_0.3 against annotation edit distance (AED).

(a)

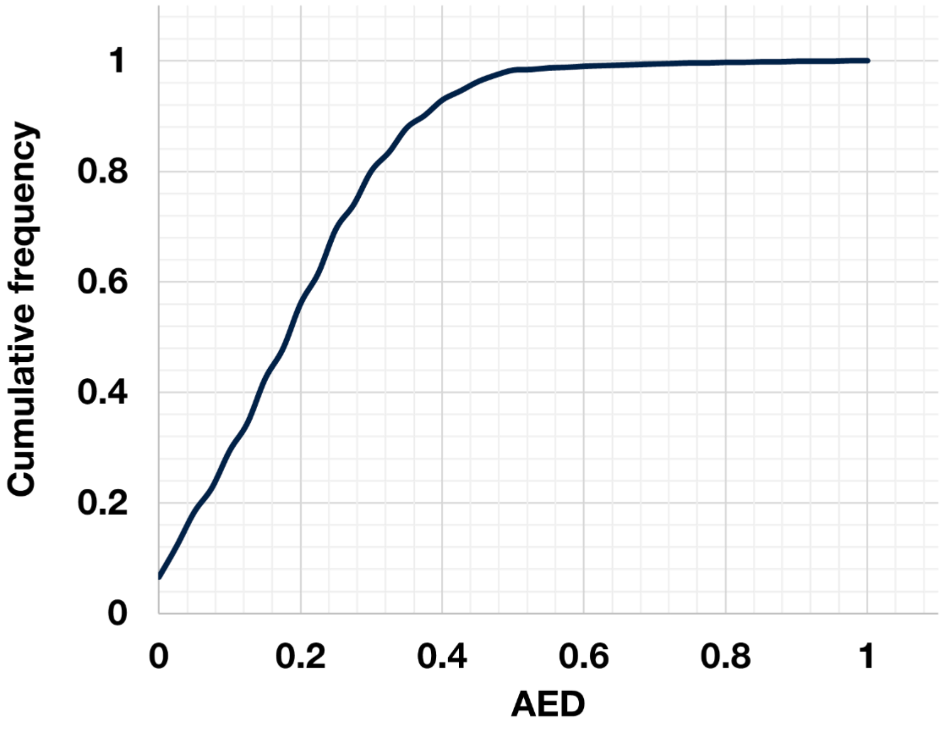

(b)

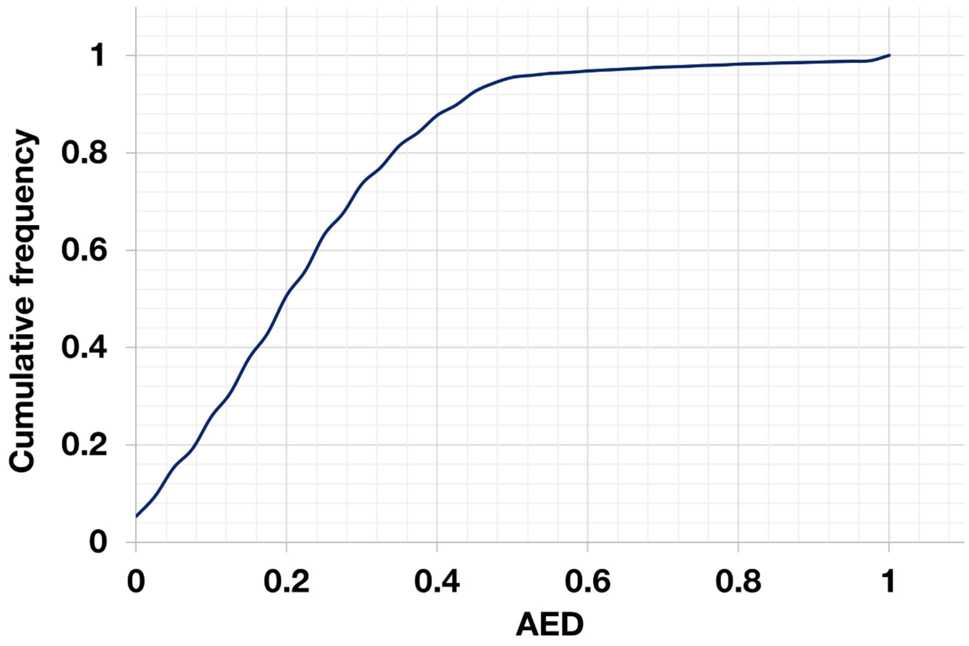

Supplementary Table 7.

Basic statistics of the ecological genomic study in *D. cochinchinensis* and *D. oliveri*.

	D. cochinchinensis	***D. oliveri***
# Samples	435	331
# Localities	35	28
# SNPs after variant calling	1,832,629	3,377,855
# SNPs after filtering for missing data and MAF	246,225	250,954
# SNPs after pruning for linkage disequilibrium	180,944	193,724
# Adaptive SNPs predicted by sNMF	50,951	54,154
# Adaptive SNPs predicted by LFMM	20,373	6,953

Supplementary Figure 3.

Cross-entropy criterion as a function of the number of factors in the sNMF analysis for **(a)** *D. cochinchinensis* and **(b)** *D. oliveri*.

(a)

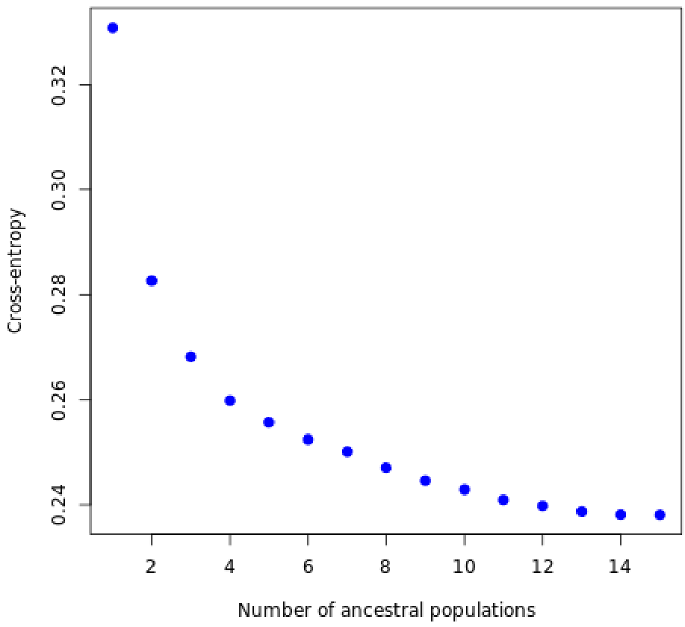

(b)

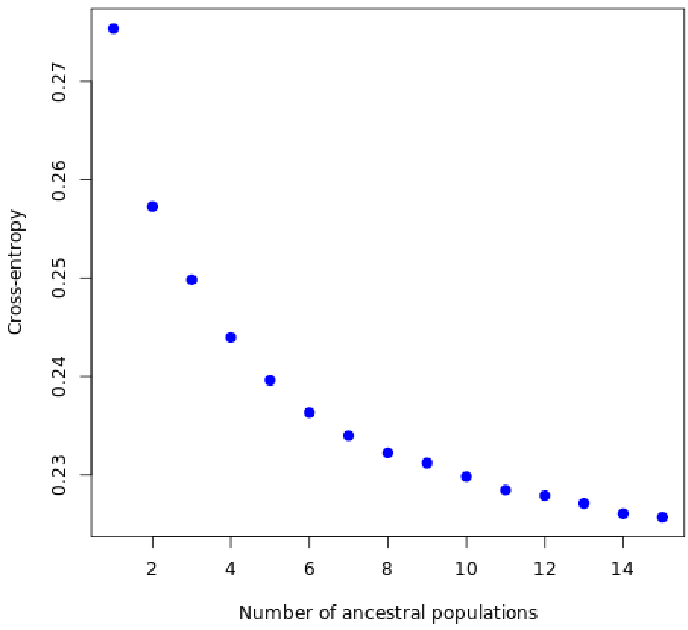

Supplementary Figure 4.

Admixture results of sNMF analysis showing hierarchical population substructure of **(a)** *D. cochinchinensis* and **(b)** *D. oliveri*.

(a)

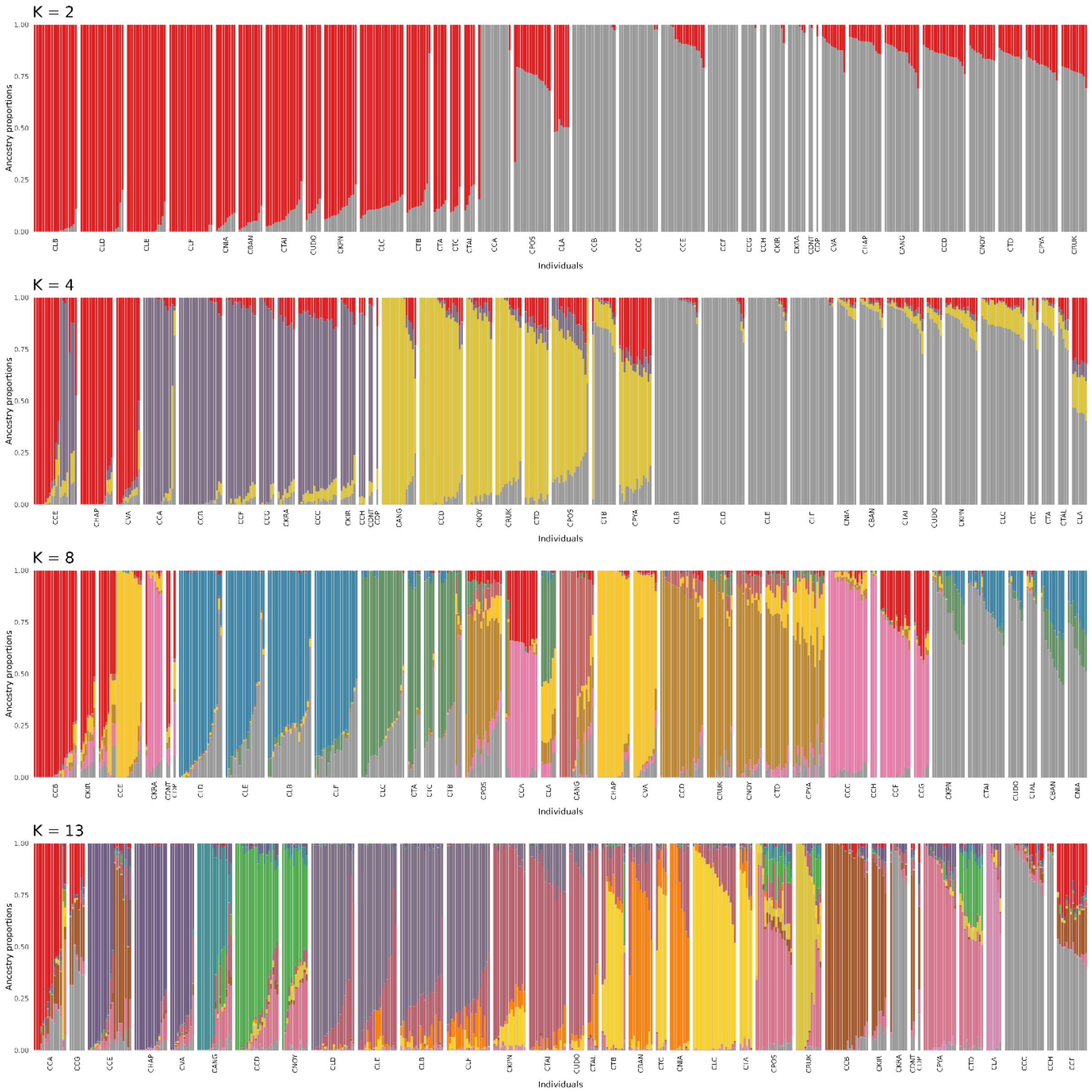

(b)

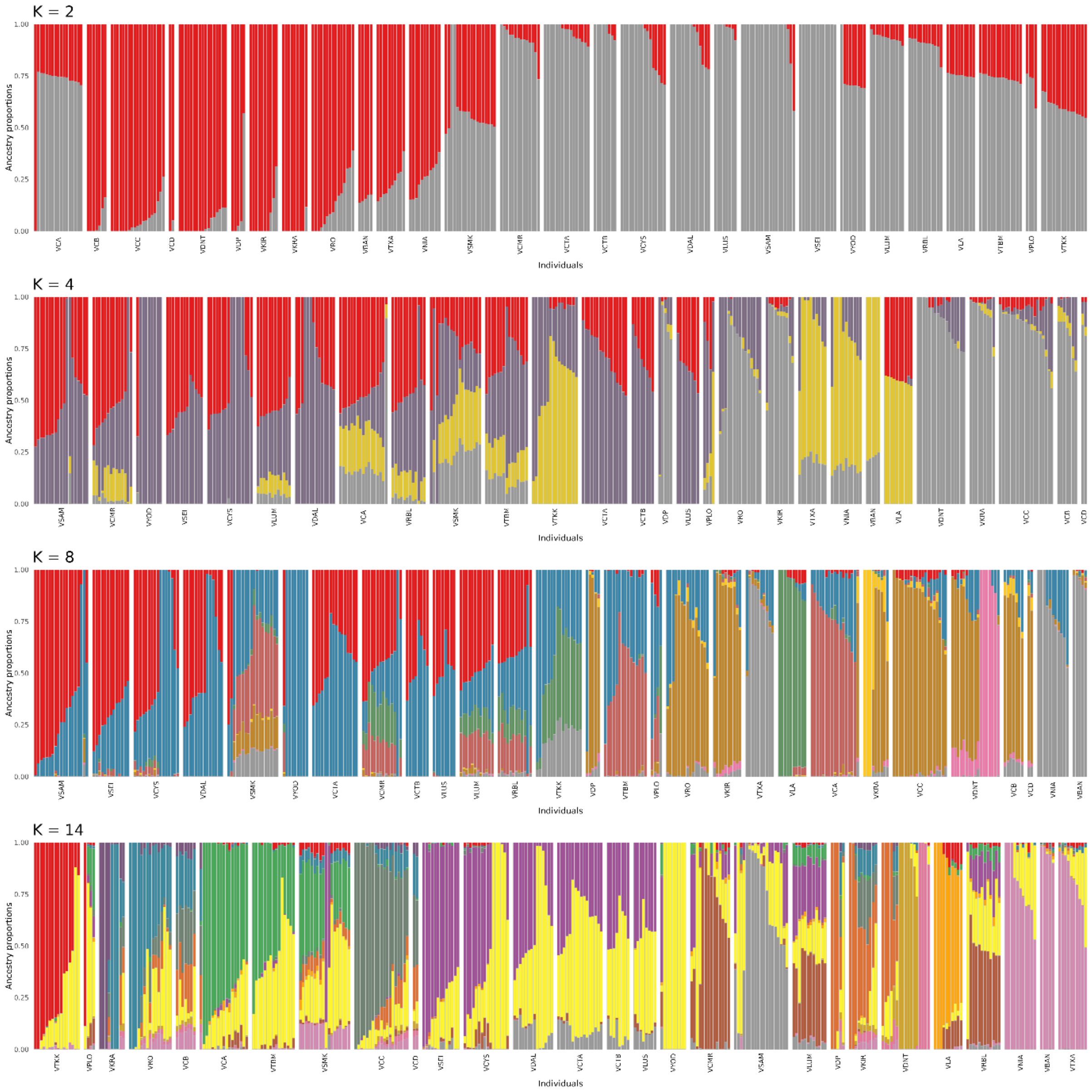
K = 2
K = 4
K = 8
K = 14
Ancestry proportions
Individuals
1.00
0.75
0.50
0.25
0.00

Supplementary Figure 5.

Admixture results of sNMF analysis showing hierarchical population substructure of **(a)** *D. cochinchinensis* and **(b)** *D. oliveri*.

(a)

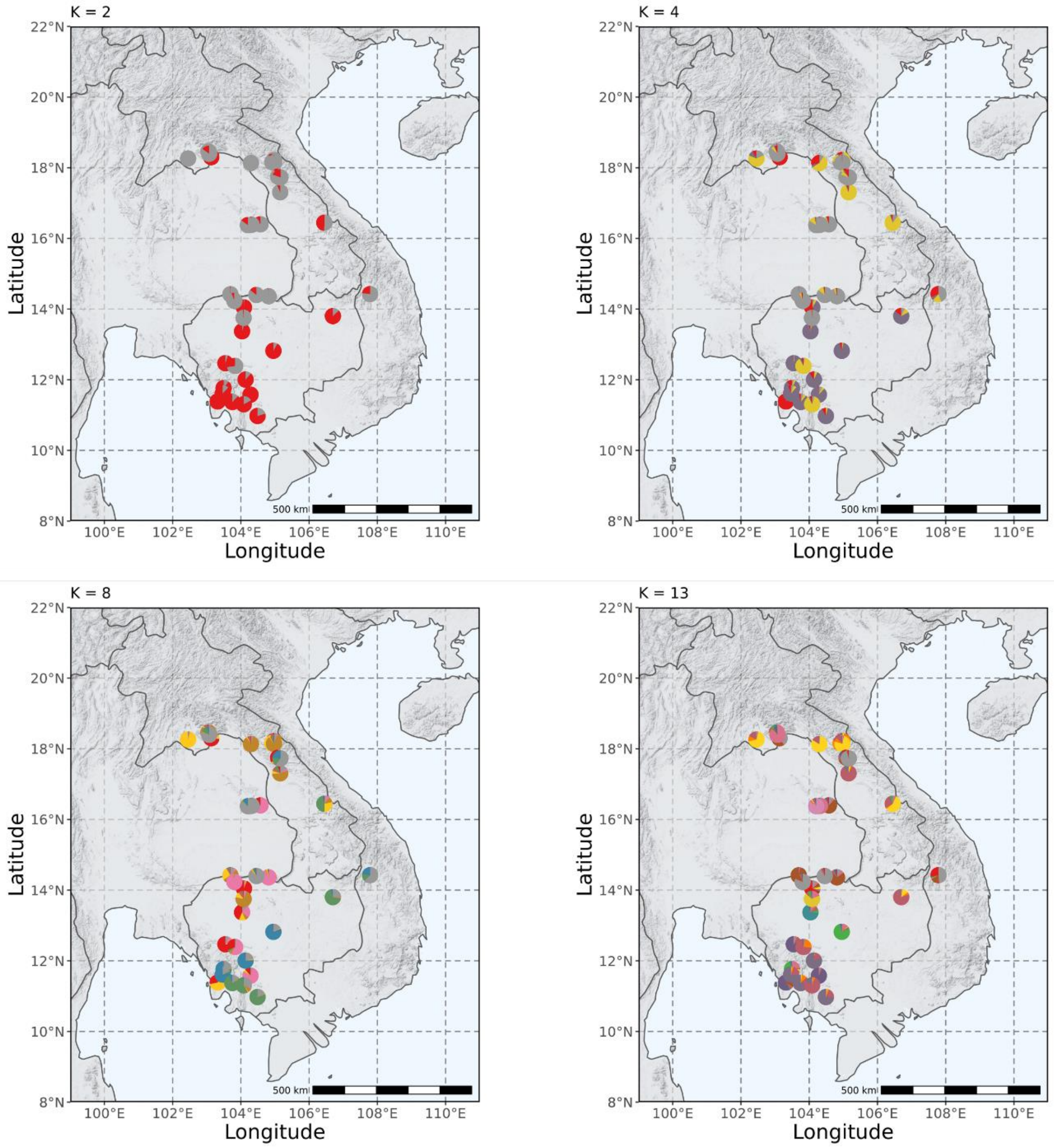

(b)

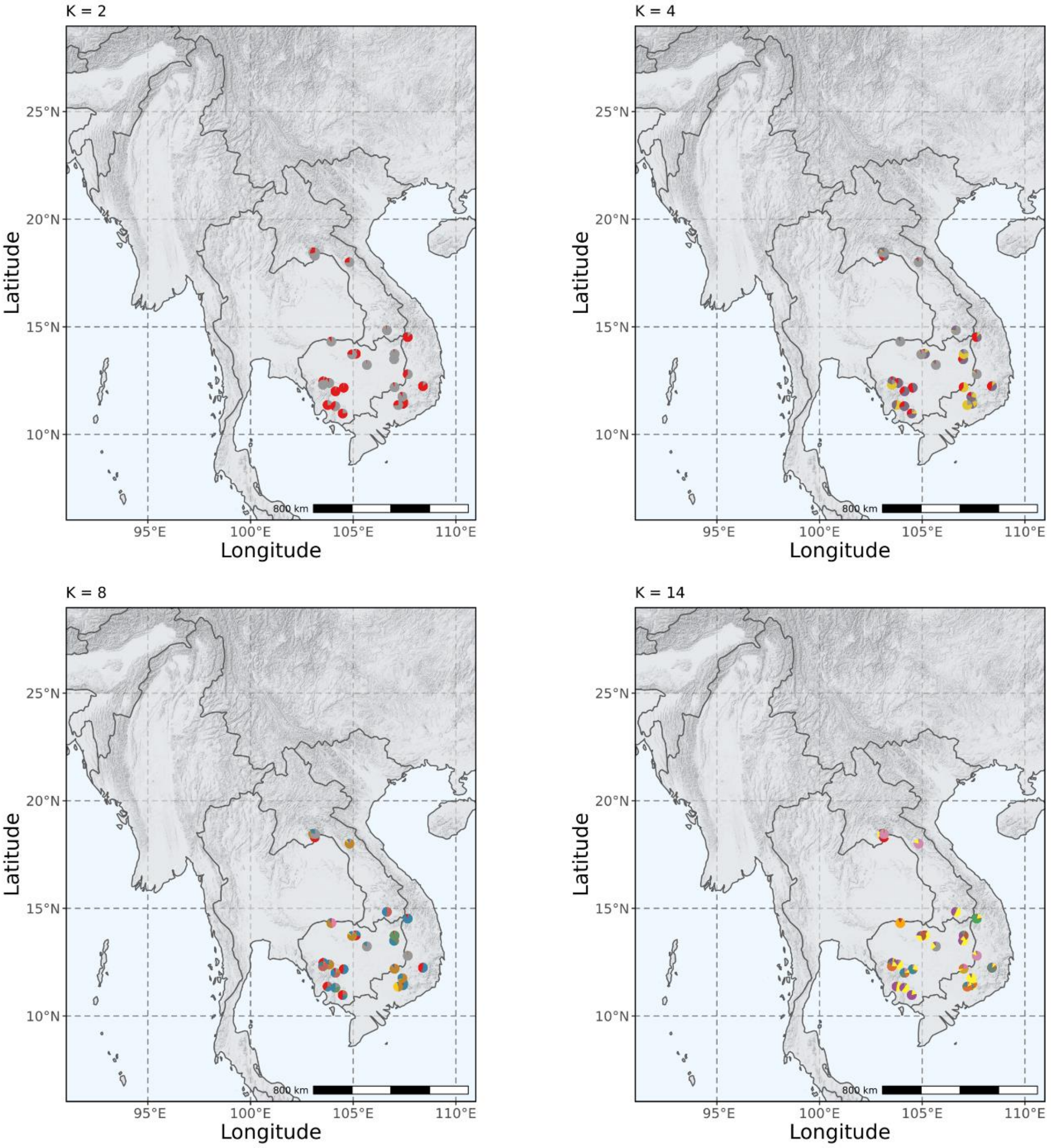
K = 2
K = 4
K = 8
K = 14
Latitude
Longitude
25°N
20°N
15°N
10°N
95°E
100°E
105°E
110°E
800 km

Supplementary Table 8.

Genomic inflation factor λ for the gene-environment associations of **(a)** *D. cochinchinensis* and **(b)** *D. oliveri*

**env**	**lambda**
evapotrans	0.071053
s_AWCh1_sl2	0.1012
s_AWCh1_sl5	0.097883
s_CLYPPT_sl2	0.0773
s_ORCDRC_sl5	0.103751
s_PHIHOX_sl5	0.11886
s_SLTPPT_sl2	0.117395
wc2.1_30s_bio_13	0.166612
wc2.1_30s_bio_17	0.247199
wc2.1_30s_bio_19	0.136046
wc2.1_30s_bio_2	0.186022
wc2.1_30s_bio_3	0.144369
wc2.1_30s_bio_8	0.088284
**mean**	**0.127383**
**sd**	**0.049323**

**env**	**lambda**
evapotrans	0.038403
npp	0.046587
s_AWCh1_sl2	0.037444
s_AWCh1_sl5	0.057747
s_ORCDRC_sl2	0.05131
s_PHIHOX_sl2	0.05708
s_SLTPPT_sl5	0.050496
s_SNDPPT_sl5	0.032456
wc2.1_30s_bio_14	0.070037
wc2.1_30s_bio_16	0.079196
wc2.1_30s_bio_2	0.080899
wc2.1_30s_bio_3	0.069888
**mean**	**0.055962**
**sd**	**0.016241**

Supplementary Figure 6.

Manhattan plot of adaptive SNPs predicted by LFMM for **(a)** *D. cochinchinensis* and **(b)** *D. oliveri*. Blue dots are adaptive SNPs for at least one environmental variable (| *Z*-value | > 2 & *Q*-value < 0.01), light-blue dots are neutral SNPs.

(a)

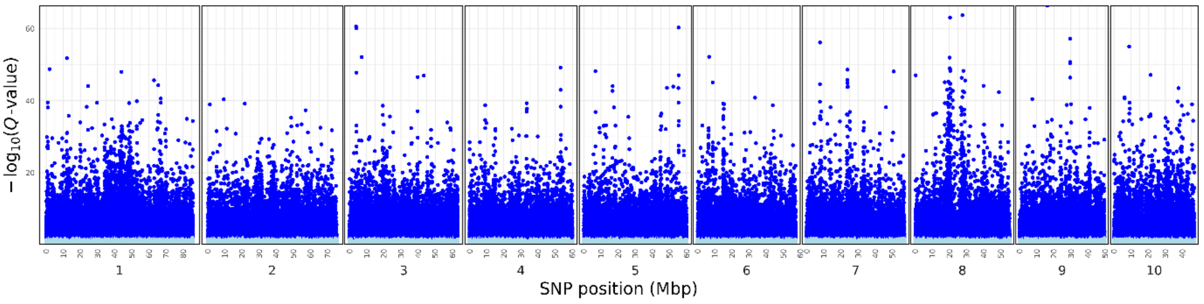

(b)

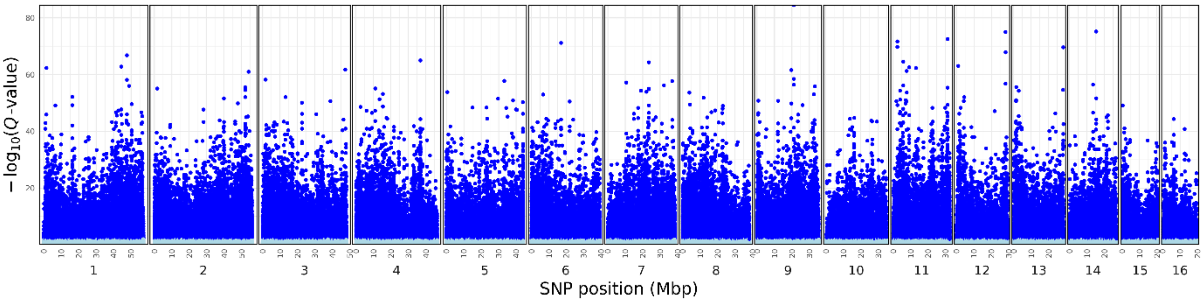

Supplementary Figure 7.

Frequency histogram of adjusted *P*-values for each environmental association of the LFMM analysis in **(a)** *D. cochinchinensis* and **(b)** *D. oliveri*.

(a)

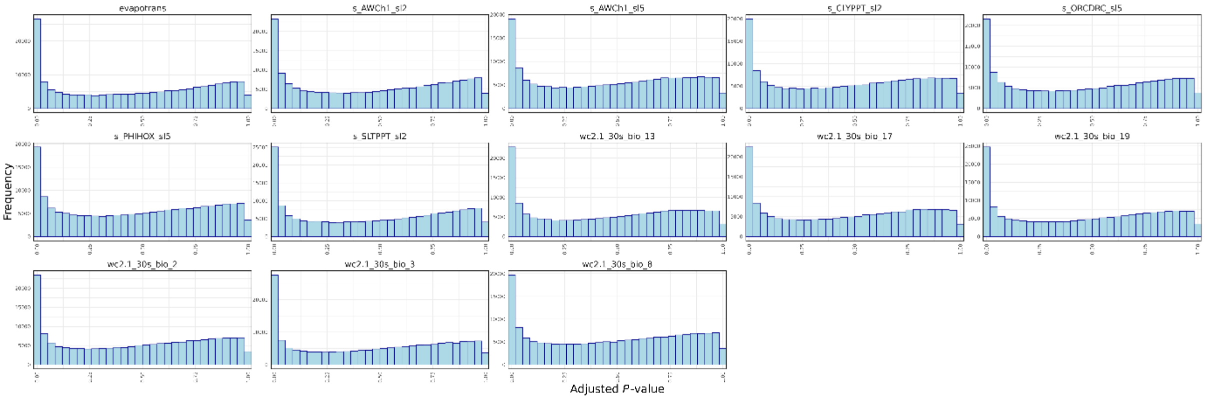

(b)

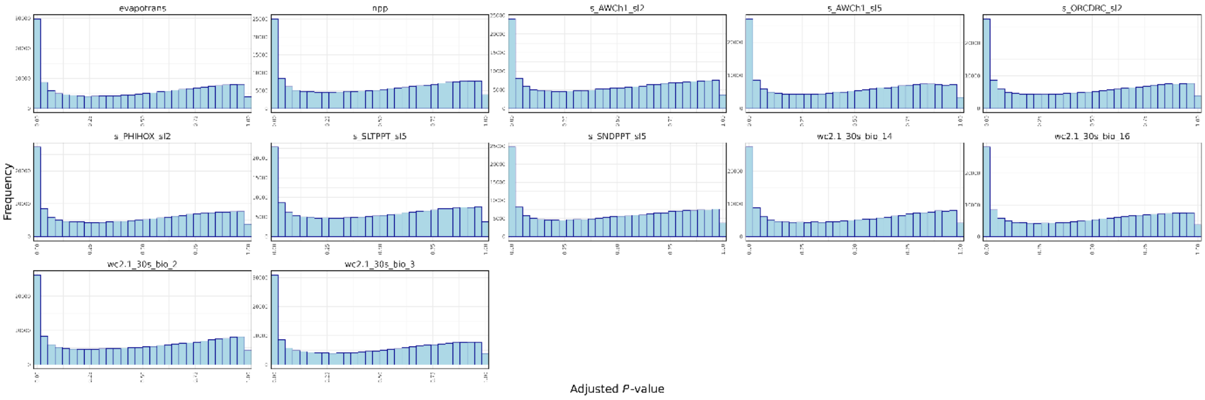

Supplementary Figure 8.

Accuracy and R^2-weighted importance for the environmental predictor variables which explained the neutral genomic variation (non-adaptive SNPs) for **(a)** *D. cochinchinensis* and **(b)** *D. oliveri* predicted by the GF model.

(a)

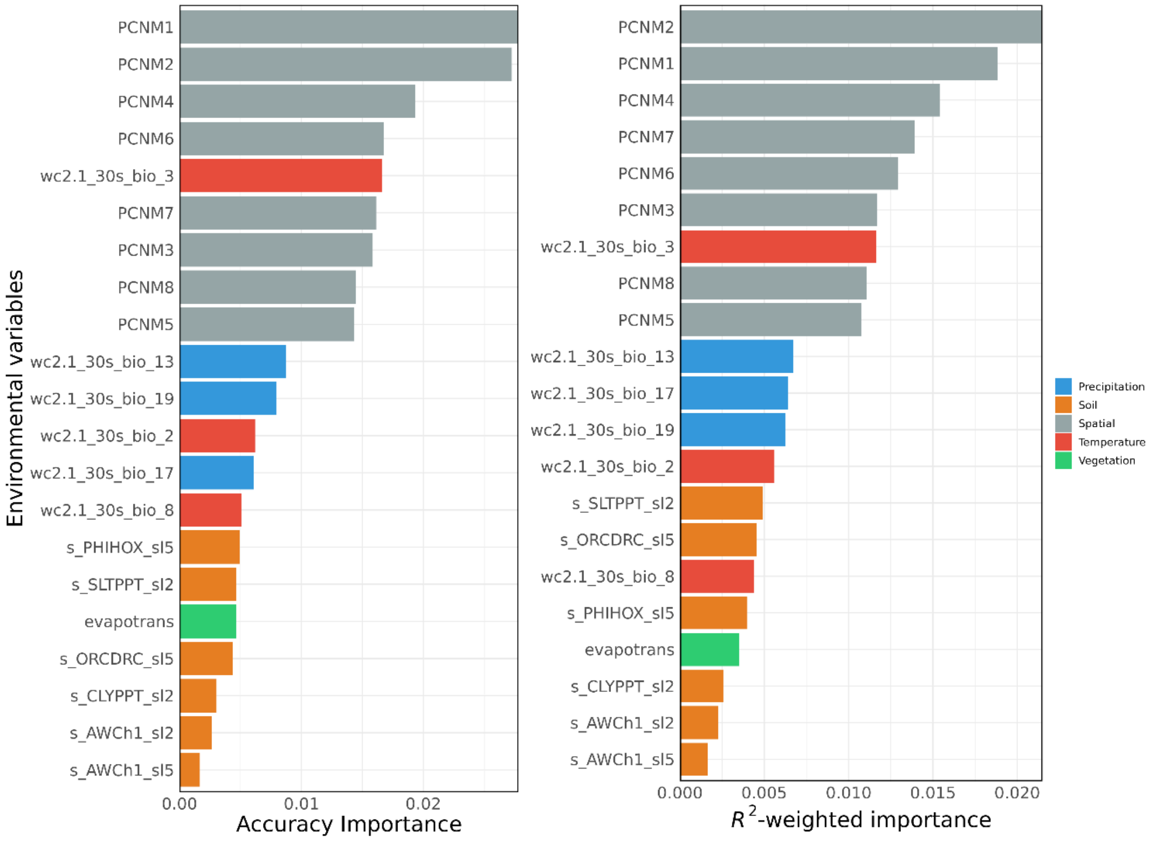

(b)

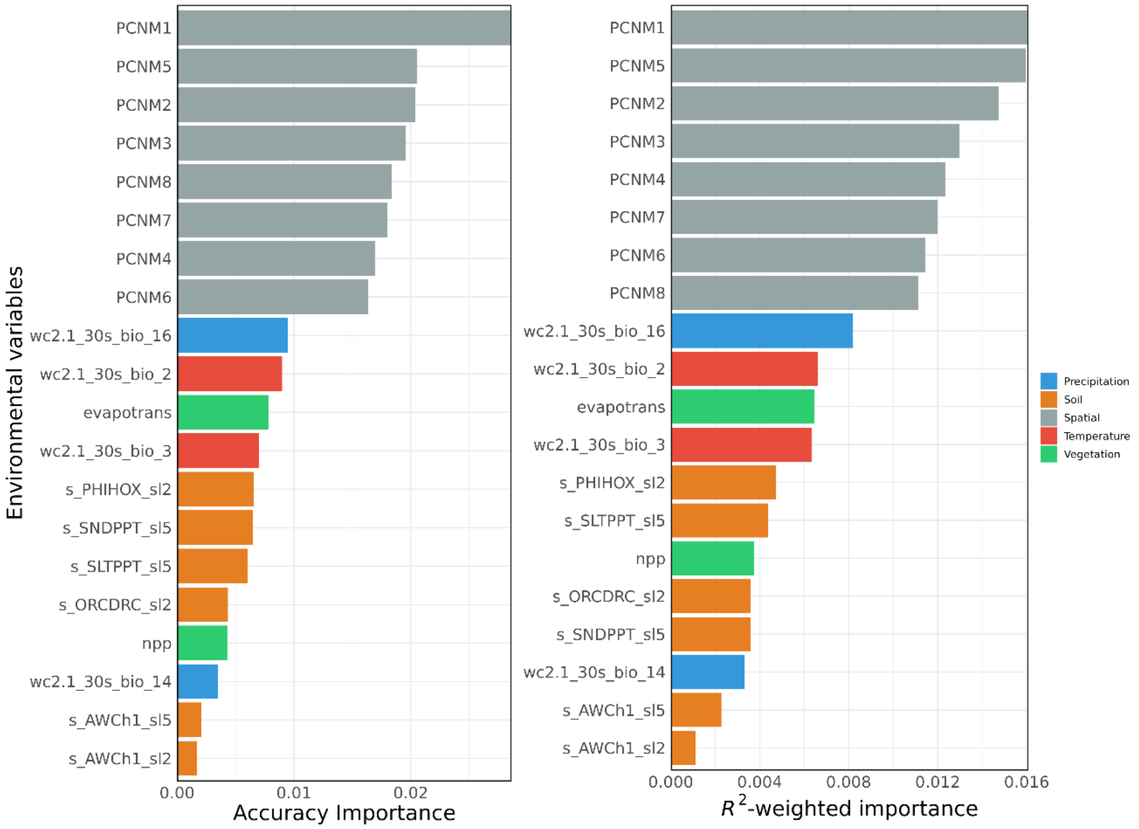

Environmental variables
PCNM1
PCNM5
PCNM2
PCNM3
PCNM8
PCNM7
PCNM4
PCNM6
wc2.1_30s_bio_16
wc2.1_30s_bio_2
evapotrans
wc2.1_30s_bio_3
s_PHIHOX_sl2
s_SNDPPT_sl5
s_SLTPPT_sl5
s_ORCDRC_sl2
npp
wc2.1_30s_bio_14
s_AWCh1_sl5
s_AWCh1_sl2
0.00
0.01
0.02
Accuracy Importance
PCNM1
PCNM5
PCNM2
PCNM3
PCNM4
PCNM7
PCNM6
PCNM8
wc2.1_30s_bio_16
wc2.1_30s_bio_2
evapotrans
wc2.1_30s_bio_3
s_PHIHOX_sl2
s_SLTPPT_sl5
npp
s_ORCDRC_sl2
s_SNDPPT_sl5
wc2.1_30s_bio_14
s_AWCh1_sl5
s_AWCh1_sl2
0.000
0.004
0.008
0.012
0.016
R^2-weighted importance
Precipitation
Soil
Spatial
Temperature
Vegetation

Supplementary Figure 9.

Allelic turnover functions of all and adaptive SNPs explaining the cumulative importance of each environmental predictor variable in **(a)** *D. cochinchinensis* and **(b)** *D. oliveri*.

(a)

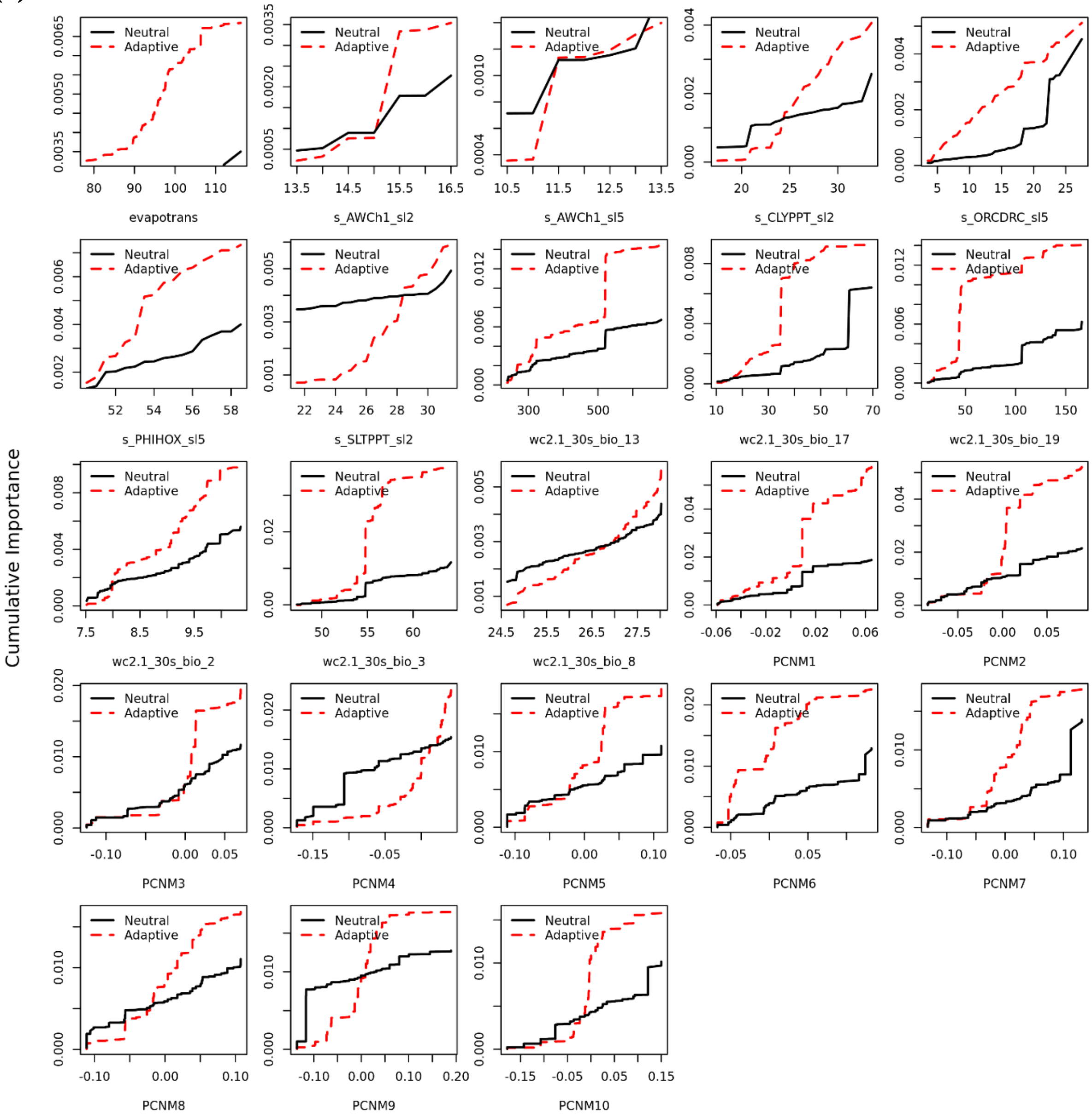

(b)

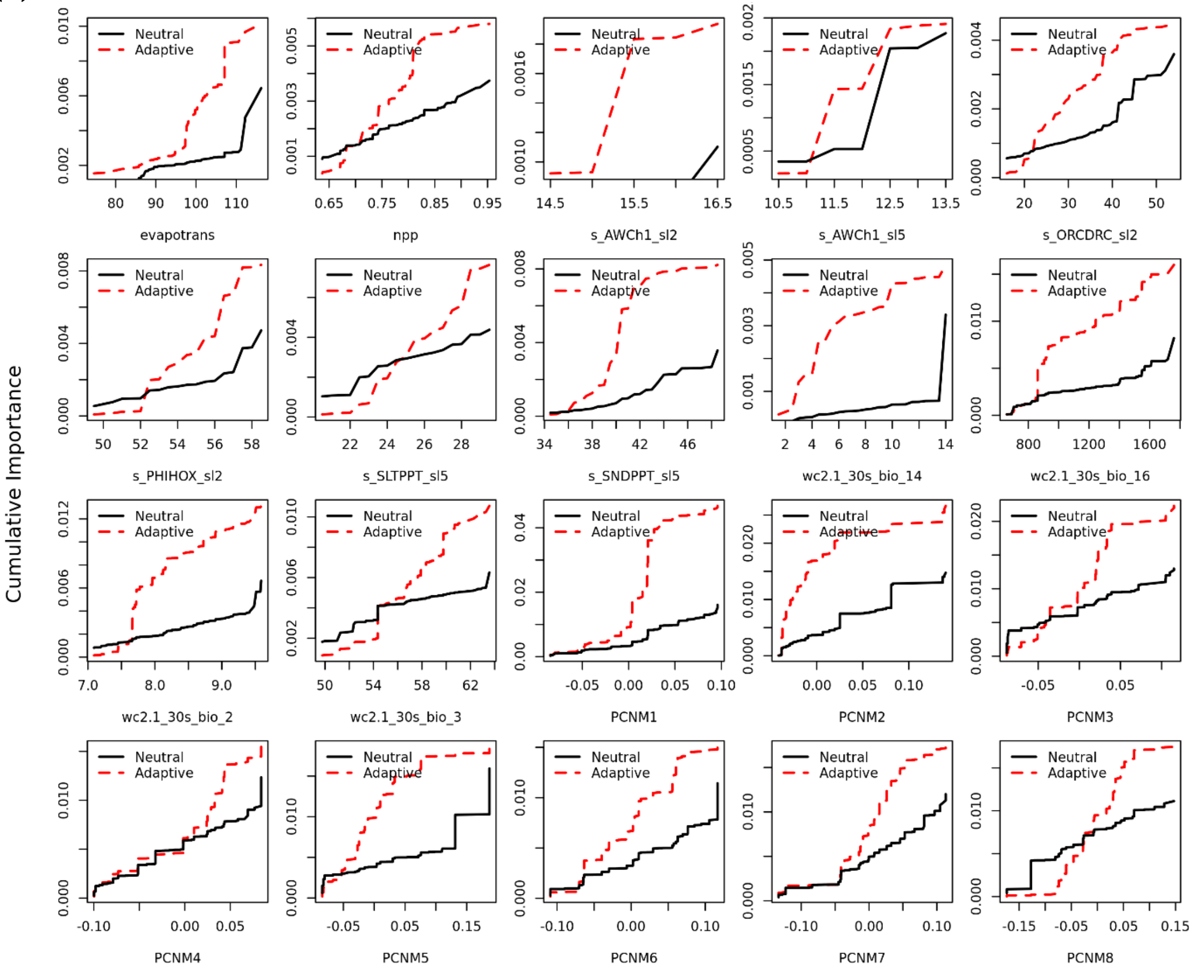
Cumulative Importance
Neutral
Adaptive
evapotrans
npp
s_AWCh1_sl2
s_AWCh1_sl5
s_ORCDRC_sl2
s_PHIHOX_sl2
s_SLTPPT_sl5
s_SNDPPT_sl5
wc2.1_30s_bio_14
wc2.1_30s_bio_16
wc2.1_30s_bio_2
wc2.1_30s_bio_3
PCNM1
PCNM2
PCNM3
PCNM4
PCNM5
PCNM6
PCNM7
PCNM8

Supplementary Figure 10.

Genetic offset of gene-environmental association (scaled between 0, lowest offset, and 1, highest offset) across the range of **(a)** *D. cochinchinensis* and **(b)** *D. oliveri* in 4 SSPs (126, 245, 370, and 585) over three bidecades (2041–2060, 2061–2080, 2081–2100) averaged across five GCMs (BCC-CSM2-MR, CNRM-ESM2-1, IPSL-CM6A-LR, MIROC6, MRI-ESM2-0.

(a)

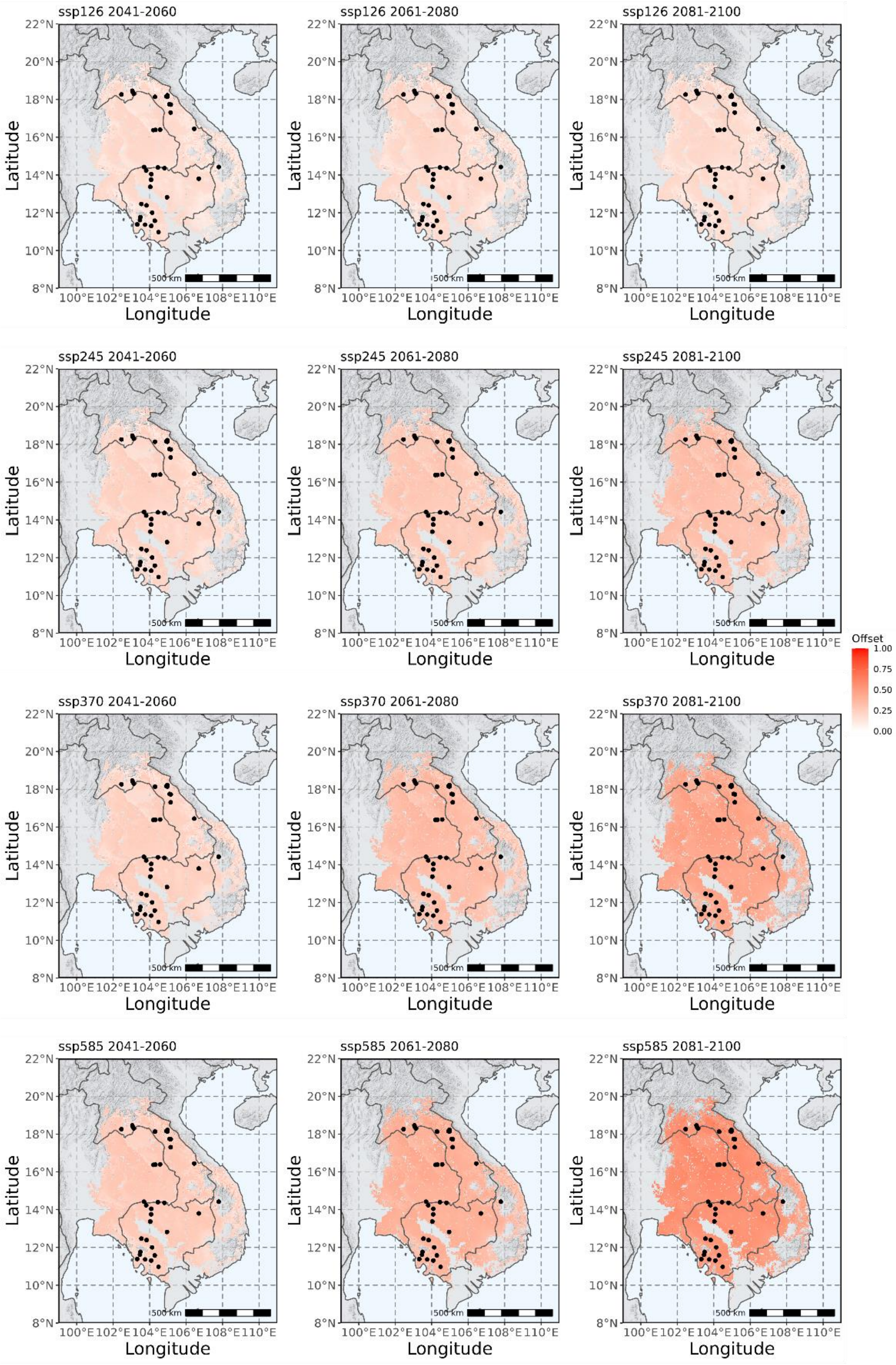

(b)

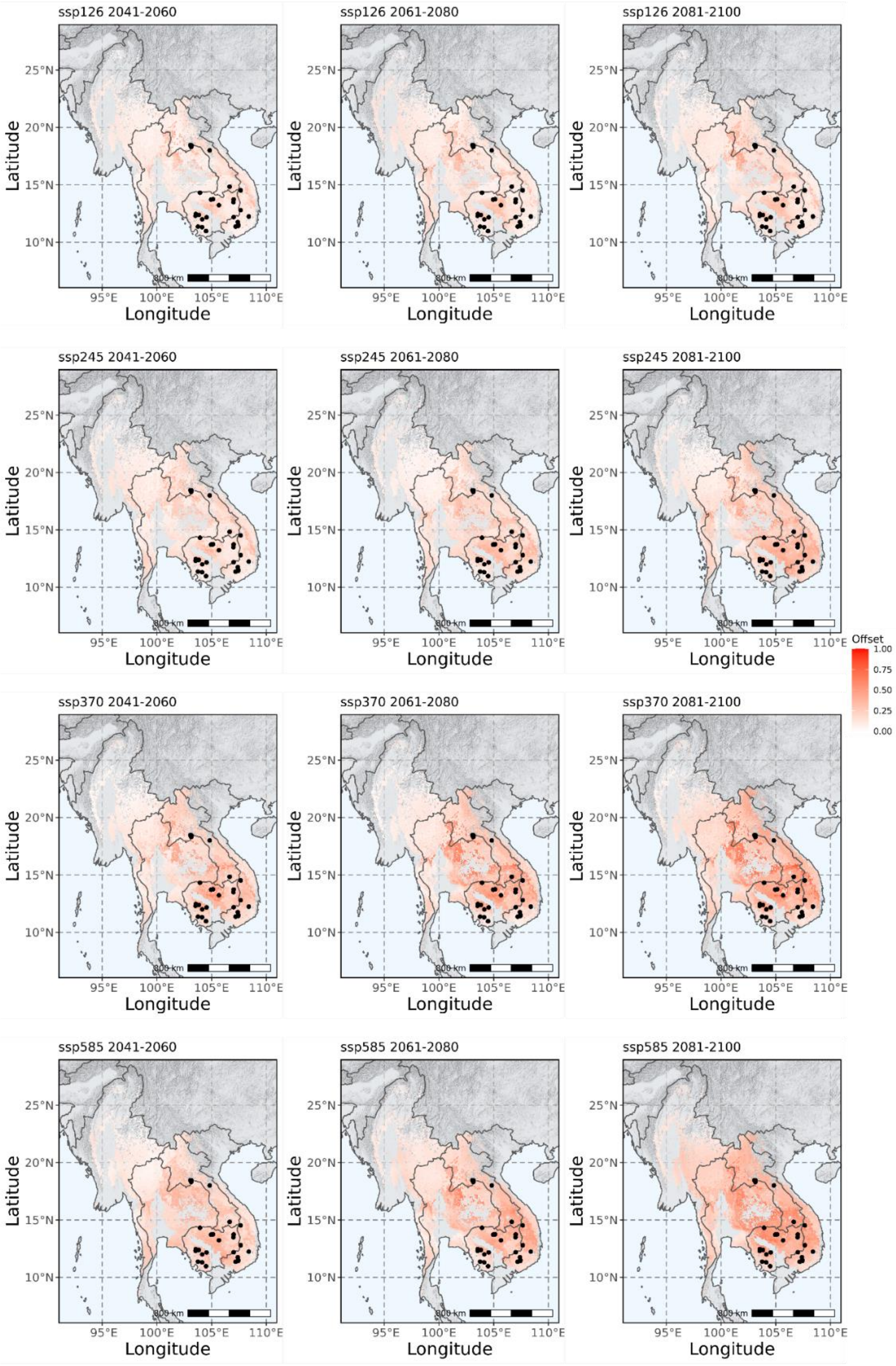
ssp126 2041-2060
ssp126 2061-2080
ssp126 2081-2100
ssp245 2041-2060
ssp245 2061-2080
ssp245 2081-2100
ssp370 2041-2060
ssp370 2061-2080
ssp370 2081-2100
ssp585 2041-2060
ssp585 2061-2080
ssp585 2081-2100
Latitude
Longitude
25°N
20°N
15°N
10°N
95°E
100°E
105°E
110°E
800 km
Offset
1.00
0.75
0.50
0.25
0.00

Supplementary Table 9.

Analysis of variance (ANOVA) table of the averaged genomic vulnerability on the interactions among time, SSP, and species.

	**Df**	**Sum Sq**	**Mean Sq**	**F-value**	**Pr (>F)**	
Time	1	2.1558e-05	2.1558e-05	48.8719	2.706e-10	***
SSP	3	3.9757e-05	1.3252e-05	30.0432	4.535e-14	***
Species	1	1.0318e-04	1.0318e-04	233.9018	< 2.2e-16	***
Time:SSP	3	9.2440e-06	3.0810e-06	6.9850	0.0002509	***
Time:Species	1	6.6060e-06	6.6060e-06	14.9762	0.0001902	***
SSP:Species	3	1.0555e-05	3.5180e-06	7.9759	7.761e-05	***
Time:SSP:Species	3	4.3060e-06	1.4350e-06	3.2542	0.0246941	*
Residuals	104	4.5876e-06	4.4100e-07			

Supplementary Table 10.

List of environmental data used in the landscape genomics modelling of this study.

Symbol	Description	Category	Source	*D. cochinchinensis*	*D. oliveri*
wc2.1_30s_bio_1	Annual mean temperature	Temperature	WorldClim		
wc2.1_30s_bio_2	Mean diurnal range	Temperature	WorldClim	V	V
wc2.1_30s_bio_3	Isothermality	Temperature	WorldClim	V	V
wc2.1_30s_bio_4	Temperature seasonality	Temperature	WorldClim		
wc2.1_30s_bio_5	Maximum temperature of warmest month	Temperature	WorldClim		
wc2.1_30s_bio_6	Minimum temperature of coldest month	Temperature	WorldClim		
wc2.1_30s_bio_7	Temperature annual range	Temperature	WorldClim		
wc2.1_30s_bio_8	Mean temperature of wettest quarter	Temperature	WorldClim	V	
wc2.1_30s_bio_9	Mean temperature of driest quarter	Temperature	WorldClim		
wc2.1_30s_bio_10	Mean temperature of warmest quarter	Temperature	WorldClim		
wc2.1_30s_bio_11	Mean temperature of coldest quarter	Temperature	WorldClim		
wc2.1_30s_bio_12	Annual precipitation	Precipitation	WorldClim		
wc2.1_30s_bio_13	Precipitation of wettest month	Precipitation	WorldClim	V	
wc2.1_30s_bio_14	Precipitation of driest month	Precipitation	WorldClim		V
wc2.1_30s_bio_15	Precipitation seasonality	Precipitation	WorldClim		
wc2.1_30s_bio_16	Precipitation of wettest quarter	Precipitation	WorldClim		V
wc2.1_30s_bio_17	Precipitation of driest quarter	Precipitation	WorldClim	V	
wc2.1_30s_bio_18	Precipitation of warmest quarter	Precipitation	WorldClim		

wc2.1_30s_bio_19	Precipitation of coldest quarter	Precipitation	WorldClim	V	
wc2.1_30s_bio_elev	Digital elevation	Elevation	WorldClim		
s_AwCh1_sl2	Available soil water capacity (volumetric fraction) with FC = pF 2.0 at depth of 5 cm	Soil	SoilGrids	V	V
s_AwCh1_sl5	Available soil water capacity at depth of 60 cm	Soil	SoilGrids	V	V
s_CLYPPT_sl2	Clay content in percent at depth of 5 cm	Soil	SoilGrids	V	
s_CLYPPT_sl5	Clay content in percent at depth of 60 cm	Soil	SoilGrids		
s_ORCDRC_sl2	Soil organic carbon stock at depth of 5 cm	Soil	SoilGrids		V
s_ORCDRC_sl5	Soil organic carbon stock at depth of 60 cm	Soil	SoilGrids	V	
s_PHIHOX_sl2	Soil pH measured in water solution at depth of 5 cm	Soil	SoilGrids		V
s_PHIHOX_sl5	Soil pH measured in water solution at depth of 60 cm	Soil	SoilGrids	V	
s_SLTPPT_sl2	Silt content in percent at depth of 5 cm	Soil	SoilGrids	V	
s_SLTPPT_sl5	Silt content in percent at depth of 60 cm	Soil	SoilGrids		V
s_SNDPPT_sl2	Sand content in percent at depth of 5 cm	Soil	SoilGrids		
s_SNDPPT_sl5	Sand content in percent at depth of 60 cm	Soil	SoilGrids		V
npp	Net primary productivity	Vegetation	The Atlas of the Biosphere		V

evapotrans	Evapotranspiration	Vegetation	The Atlas of the Biosphere	V	V

Supplementary Figure 11.

The user interface of the "Predict" function of *seedeR* (https://github.com/hung-th/seedeR). In the left panel "Inputs", the user can choose a species, a SSP, a time period and input the longitude and the latitude of the future restoration site. After clicking "Run", the process will begin and print the output In the right panel "Output".

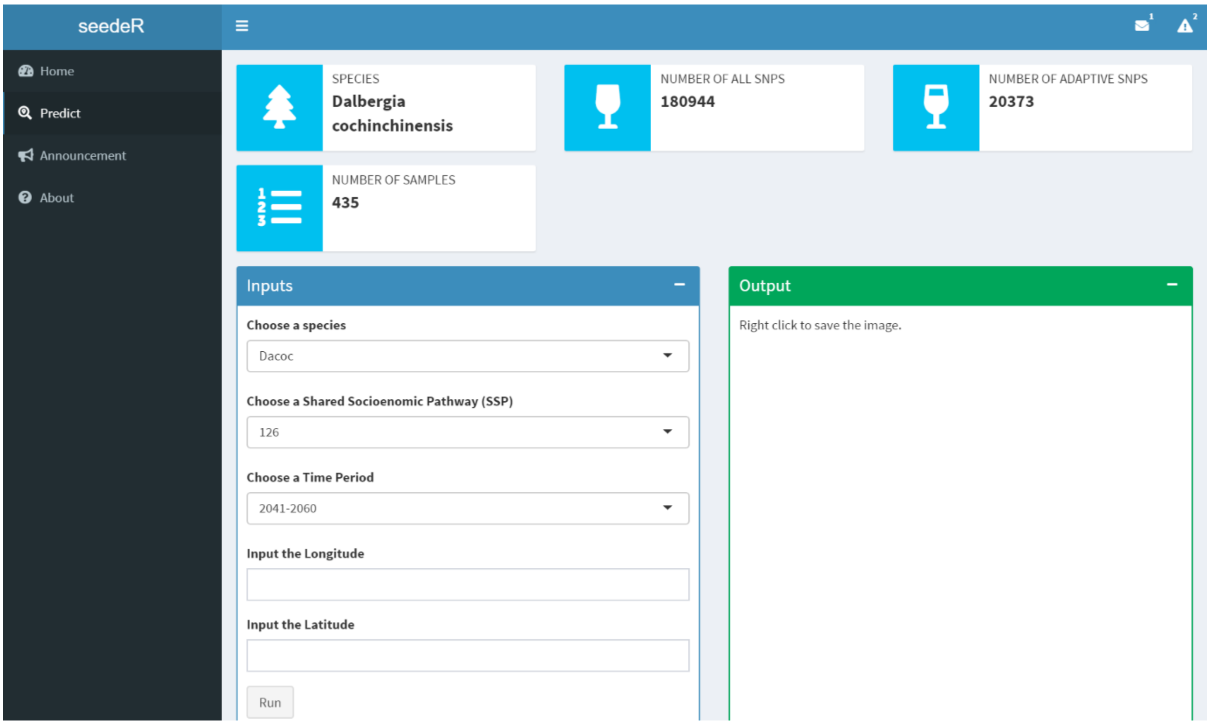

Supplement: Supplementary file 1 — Appendix 01 (PDF) [file pnas.2301603120.sapp.pdf]
